# Event-Associated Oxygen Consumption Rate Increases ca. Five-Fold When Interictal Activity Transforms into Seizure-Like Events In Vitro

**DOI:** 10.3390/ijms18091925

**Published:** 2017-09-07

**Authors:** Karl Schoknecht, Nikolaus Berndt, Jörg Rösner, Uwe Heinemann, Jens P. Dreier, Richard Kovács, Alon Friedman, Agustin Liotta

**Affiliations:** 1 Department of Experimental Neurology, Charité–Universitätsmedizin Berlin, Corporate Member of Freie Universität Berlin, Humboldt-Universität zu Berlin, and Berlin Institute of Health, 10117 Berlin, Germany; karl.schoknecht@charite.de (K.S.); jens.dreier@charite.de (J.P.D.); 2 Center for Stroke Research Berlin, Charité–Universitätsmedizin Berlin, Corporate Member of Freie Universität Berlin, Humboldt-Universität zu Berlin, and Berlin Institute of Health, 10117 Berlin, Germany; 3 Neuroscience Research Center, Charité–Universitätsmedizin Berlin, Corporate Member of Freie Universität Berlin, Humboldt-Universität zu Berlin, and Berlin Institute of Health, 10117 Berlin, Germany; joerg.roesner@charite.de (J.R.); karl.schoknecht@googlemail.com (U.H.); 4 Institute of Biochemistry, Charité–Universitätsmedizin Berlin, Corporate Member of Freie Universität Berlin, Humboldt-Universität zu Berlin, and Berlin Institute of Health, 10117 Berlin, Germany; nikolaus.berndt@charite.de; 5 Department of Neurology, Charité–Universitätsmedizin Berlin, Corporate Member of Freie Universität Berlin, Humboldt-Universität zu Berlin, and Berlin Institute of Health, 10117 Berlin, Germany; 6 Institute for Neurophysiology, Charité–Universitätsmedizin Berlin, Corporate Member of Freie Universität Berlin, Humboldt-Universität zu Berlin, and Berlin Institute of Health, 10117 Berlin, Germany; richard.kovacs@charite.de; 7 Department of Medical Neuroscience, Faculty of Medicine, Dalhousie University, Halifax, NS B3H 4R2, Canada; alon.friedman@Dal.Ca; 8 Departments of Physiology and Cell Biology, Cognitive and Brain Sciences, Zlotowski Center for Neuroscience, Ben-Gurion University of the Negev, 84105 Beer-Sheva, Israel; 9 Department of Anesthesiology and Intensive Care, Charité–Universitätsmedizin Berlin, Corporate member of Freie Universität Berlin, Humboldt-Universität zu Berlin and Berlin Institute of Health, 10117 Berlin, Germany; 10 Berlin Institute of Health (BIH), Anna-Louisa-Karsch-Str. 2, 10178 Berlin, Germany

**Keywords:** epilepsy, oxygen, ATP

## Abstract

Neuronal injury due to seizures may result from a mismatch of energy demand and adenosine triphosphate (ATP) synthesis. However, ATP demand and oxygen consumption rates have not been accurately determined, yet, for different patterns of epileptic activity, such as interictal and ictal events. We studied interictal-like and seizure-like epileptiform activity induced by the GABA_A_ antagonist bicuculline alone, and with co-application of the M-current blocker XE-991, in rat hippocampal slices. Metabolic changes were investigated based on recording partial oxygen pressure, extracellular potassium concentration, and intracellular flavine adenine dinucleotide (FAD) redox potential. Recorded data were used to calculate oxygen consumption and relative ATP consumption rates, cellular ATP depletion, and changes in FAD/FADH_2_ ratio by applying a reactive-diffusion and a two compartment metabolic model. Oxygen-consumption rates were ca. five times higher during seizure activity than interictal activity. Additionally, ATP consumption was higher during seizure activity (~94% above control) than interictal activity (~15% above control). Modeling of FAD transients based on partial pressure of oxygen recordings confirmed increased energy demand during both seizure and interictal activity and predicted actual FAD autofluorescence recordings, thereby validating the model. Quantifying metabolic alterations during epileptiform activity has translational relevance as it may help to understand the contribution of energy supply and demand mismatches to seizure-induced injury.

## 1. Introduction

Due to its high energy demand, the brain receives about 15% of the heart stroke volume [1]. Epileptic seizures, i.e., paroxysmal, hypersynchronous, and excessive cell firing, elevate energy consumption due to massive neurotransmitter release and associated energy-dependent neurotransmitter recycling and recovery of ion gradients (largely by the Na^+^–K^+^-ATPase) [2,3]. In addition to seizures (i.e., ictal events,) brief interictal discharges can be observed in the whole cortex or in epileptic foci [4] and may result in local vasodilation suggesting increased energy demand [5]. Ivanov et al. recently described different metabolic “footprints” for ictal and interictal events in the immature mice whole hippocampus preparation—all showing different degrees of elevated metabolism indicated by drops in tissue partial pressure of oxygen (pO_2_) and changes in NADH/NAD^+^ (nicotinamide adenine dinucleotide) and FADH_2_/FAD (flavine adenine dinucleotide) ratio [6]. Positron emission tomography studies in humans often show ictal hypermetabolism and interictal hypometabolism within epileptic foci related to mitochondrial dysfunction [7,8]. Although a mismatch in energy supply and demand during and following epileptic discharges may subsequently lead to injury [9], very little is known on the actual oxygen consumption rates in relation to the energy demand during different forms of epileptic activity.

Here we describe oxygen and ATP consumption rates in two well-characterized types of epileptic activity in the hippocampal area CA3: interictal-like events (ILEs) and seizure-like events (SLEs). ILEs were pharmacologically induced by the gamma aminobutyric acid receptor A (GABA_A_-R) antagonist bicuculline. These short hypersynchronous events, also called recurrent epileptiform discharges [10], are electrophysiologically similar to interictal spikes recorded in vivo. Compared with ILEs, SLEs are typically prolonged and associated with repetitive field potential discharges and greater increases in extracellular potassium ([K^+^]_o_) [11,12]. To induce SLEs in the same preparation, we co-applied bicuculline and the M-current blocker XE-991. M-currents are non-inactivating outward potassium currents mediated by Kv7 channels that stabilize the resting membrane potential [13]. Importantly, Kv7 channel mutations were found in epilepsy syndromes [14,15].

To assess cerebral metabolism of ILEs and SLEs, we simultaneously recorded local field potential, ([K^+^]_o_), pO_2_ and FAD autofluorescence. Kinetics of [K^+^]_o_ reflect the ion movements during neuronal de- and repolarization, while changes in tissue pO_2_—in the absence of blood flow—reflect cellular respiration. As FAD emits fluorescence when excited at 450 nm (FADH_2_ is non-fluorescent), changes in the autofluorescence (>515 nm) give an indication of the redox changes in the FADH_2_/FAD couple [6,16,17]. Using a model of oxygen transport and consumption within brain slice preparations [18] and measured pO_2_ depth profiles, we calculated oxygen consumption rates (OCRs) during ILEs and SLEs. Subsequently, ATP consumption rates, intracellular ATP levels and FAD reduction states were calculated from the OCRs by applying a model of neuronal energy metabolism [19]. Model predictions were, in part, validated by comparing computed FAD reduction states with recorded FAD autofluorescence signals.

## 2. Results

### 2.1. Properties of Interictal- and Seizure-Like Events in Hippocampal Area CA3

**Figure 1:**
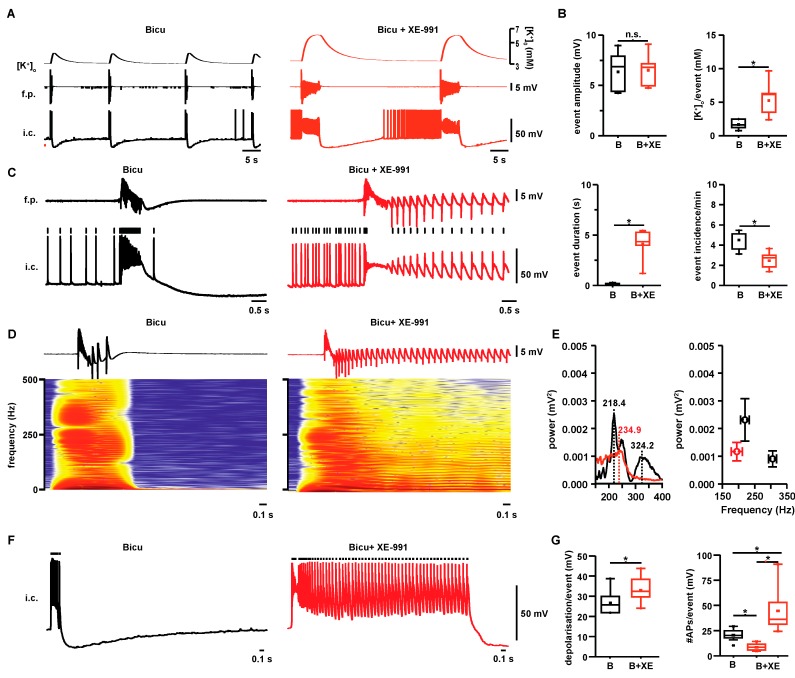
Properties of ILEs and SLEs: (**A**) Simultaneous recording of field potential (f.p.), extracellular potassium concentration ([K^+^]_o_) and intracellular recording from CA3 pyramids (i.c., bottom traces) during interictal-like events (ILEs) induced by bicuculline (black) and seizure-like events (SLEs) induced by the co-application of bicuculline and XE-991 (red); (**B**) ILEs and SLEs differed significantly in event-associated [K^+^]_o_-rises, event duration and incidence, while event amplitudes remained unaltered (* *p* < 0.05, *n* = 6, paired *t*-test); (**C**) Detail of typical f.p. recording and i.c. correlate during ILEs and SLEs. Bicuculline-induced ILEs are short paroxysmal discharges characterized by a slow wave with superimposed high-frequency oscillations (HFOs). The simultaneous i.c., recording shows a typical depolarization with cell firing. SLEs are characterized by an initial ILE followed by repetitive brief bursts. Compared with ILEs, cell firing is reduced during the first component (slow wave) of the discharge with concomitantly increased cell firing during the after discharges; (**D**) and (**E**) Fast Fourier transform (FFT)-based frequency sonograms of exemplary ILE and SLE. During bicuculline-induced ILE, HFOs typically displayed two main peaks (see corresponding power spectrum in **E**, black line). During SLEs, the faster oscillatory peak was abolished (red line). Right: Averaged main frequency and power of HFOs during ILEs (black) and SLEs (red) (*n* = 6); (**F**) I.c. recording during ILEs (black trace) and SLEs (red trace): bicuculline bursts are marked membrane depolarizations with high frequency action potential (AP)-firing (small black line on top). SLEs also begin with a pronounced membrane depolarization, however, with less firing, followed by repeated cell bursting; and (**G**) Compared with ILEs (black), SLEs (red) were associated with increased membrane depolarization amplitudes (left). The number of APs decreased significantly during the initial burst under SLEs (red box, middle) compared with ILEs. When the total number of APs during the whole discharge was measured (red box on the right), SLEs were associated with a marked increase in neuronal firing compared with ILEs. (* *p* < 0.05, *n* = 11 cells from six slices, paired *t*-test).

**Figure 2:**
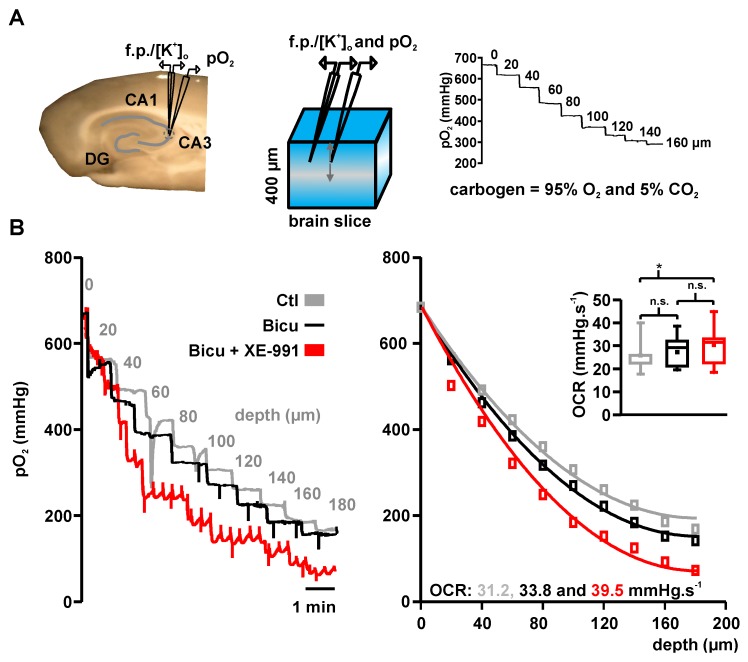
Variations in basal pO_2_ under bicuculline and bicuculline + XE-991: (**A**) Left: Picture and scheme of typical electrode placement in acute hippocampal slice. Double-barreled ion-sensitive microelectrodes (with field potential and ion-sensitive recording side) and Clark-style oxygen electrodes were placed ~80 µm below the slice surface in the pyramidal layer of area CA3. Acute brain slices receive oxygen from surface and bottom and the partial oxygen pressure (pO_2_) decreases with the distance to the source of oxygen (i.e., distance to the slice bottom and surface) providing typical depth profiles (see right, numbers in trace—distance to slice surface). The oxygen gradient along the slice depends on oxygen supply and solubility, which are both constant under experimental conditions. Hence, changes in pO_2_ depth profiles reflect activity-dependent O_2_ consumption; and (**B**) Using an established reaction-diffusion model, peak pO_2_-levels at each vertical step in the pO_2_ profile (**left**) were fitted (**right**) to calculate oxygen consumption rates (OCR). OCRs increased from control (spontaneous activity, **gray**, 31.2 mmHg·s^−1^) to induced ILEs (**black**, 33.8 mmHg·s^−1^) and SLEs (**red**, 39.5 mmHg·s^−1^) in this example. The quantitative analysis (inset) revealed a significant OCR increase during SLEs (**red**) compared with control conditions (**gray**, * *p* = 0.008, n.s. not significant, *n* = 8, paired *t*-test with Bonferroni post-hoc correction).

Two types of epileptiform activity, ILEs and SLEs were recorded in the rat CA3 hippocampal region in the acute slice preparation (Figure 1 and Figure 2A). ILEs were usually recorded within ~40 min after application of bicuculline and were characterized by paroxysmal slow wave discharges. ILEs lasted 0.18 s (0.16, 0.20; data shown as median and 25th and 75th percentile in brackets; *n* = 6) and were significantly prolonged by the additional application of XE-991 to 4.3 s (4.0, 5.3; *n* = 6, *p* < 0.05; Figure 1B). These second long paroxysmal events occurred within 20–40 min following XE-991 application and were considered SLEs. SLEs started with a burst of action potentials (resembling ILEs) followed by a succession of after-discharges (Figure 1C,D) Event amplitudes of 6.3 mV (4.4, 7.9) for ILEs were similar to 6.6 mV (5.0, 7.2) for SLEs, while the event incidence decreased significantly from 4.9 (3.6, 5.1) to 2.4 events/min (1.8, 3.0; *n* = 6, *p* < 0.05; Figure 1B). [K^+^]_o_-rises during SLEs were prolonged and elevated to 7.9 mM (6.5, 9.3; *n* = 6, *p* < 0.05, Figure 1A,B) compared with 4.5 mM (4.2, 5.5; see Figure 1B; line—median, dot—mean) during ILEs. Intracellular recordings revealed that CA3 pyramidal cells depolarized by 36.0 mV (31.4, 53.1; Figure 1G) during SLEs, which is a 24% increase compared with ILEs that depolarized by 24.9 mV (21.9, 30.0; *n* = 11 cells from six slices, *p* < 0.05). While the number of action potentials (APs) for SLEs decreased during the initial burst (7.7, range 5.8, 11.5 APs /events compared with 20.3, range 17.9, 24.9, for ILEs; *n* = 11 cells from six slices, *p* < 0.05), additional APs were evoked during SLE repetitive after-discharges (median 36.0, 31.4, 53.1 APs/event; *p* < 0.05, Figure 1F,G). High-frequency oscillations (HFOs) during ILEs were previously described [10] and typically showed two peaks at ~190 and ~290 Hz (Figure 1D,E). Compared with ILEs, HFOs during SLEs showed only one peak with lower frequency (~180 Hz; Figure 1E).

### 2.2. Basal Oxygen Consumption Rates under Bicuculline and Bicuculline Plus XE-991

To calculate oxygen consumption rates (OCRs), we applied a mathematical reaction-diffusion model [18] to experimentally recorded pO_2_ depth profiles (Figure 2A). Under interface recording conditions, pO_2_ was maximal at the slice surface (~680 mmHg) and minimal (~300 mmHg) in the slice core at a depth of ~200 μm. After trespassing the core, pO_2_ increased again as oxygen diffused from the bottom via the perfused artificial cerebrospinal fluid (aCSF). Depth profiles were recorded under control conditions (aCSF only) and in the presence of bicuculline (while recording ILEs) or bicuculline + 10 µM XE-991 (while recording SLEs; Figure 2B, also see methods). Basal pO_2_ levels during event-free intervals were used to calculate basal OCRs. Basal OCR slightly increased from 25.1 mmHg·s^−1^ (22.4, 25.8) under control conditions to 26.2 mmHg·s^−1^ (21.2, 32.0, *n* = 8) under bicuculline. When XE-991 was added to the aCSF, basal OCR significantly increased to 30.9 mmHg·s^−1^ (22.6, 33.0) (Figure 2B inset, line—median, dot—mean, *n* = 8, *p* < 0.008, paired *t*-test with Bonferroni post-hoc test).

### 2.3. Event-Associated Oxygen Consumption Rates for ILEs and SLEs

**Figure 3:**
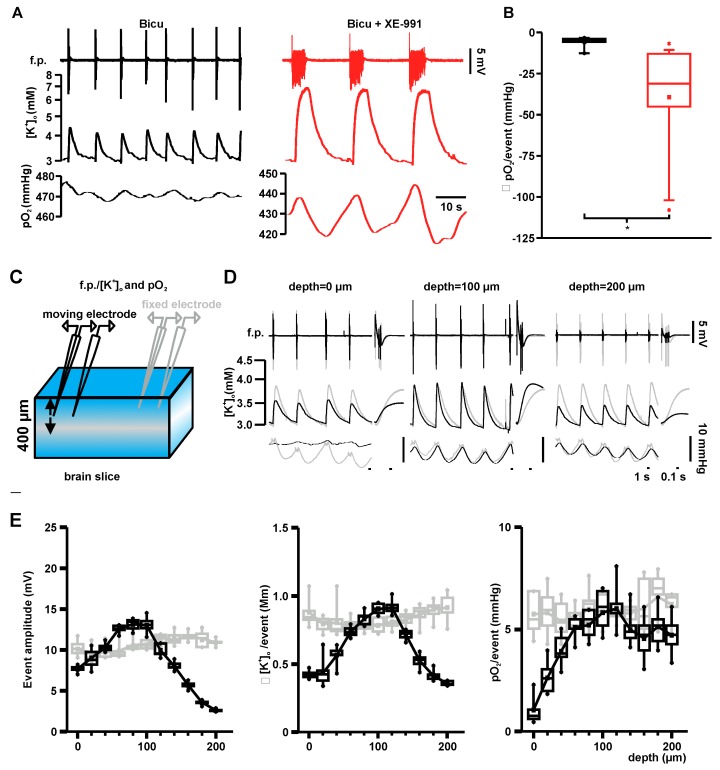

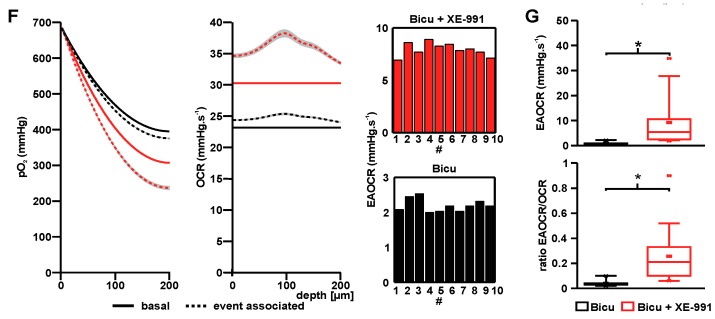
Locality of activity and modeling of local oxygen consumption rates associated with ILEs and SLEs in brain slices: (**A**) Typical f.p., extracellular potassium ([K^+^]_o_) and oxygen recordings of ILEs (**black**) and SLEs (**red**). Event-associated oxygen baseline drops were larger in SLEs compared with ILEs; (**B**) Local oxygen drops during ILEs and SLEs at 80 µm from the slice surface. Event-related pO_2_ drops during SLEs (**red**) were significantly larger compared with ILEs (**black**) (*p* = 0.011, *n* = 8 and 15 for ILEs and SLEs respectively, independent *t*-test); (**C**) Scheme of brain slice recordings under interface conditions with two pairs of double-barreled potassium-sensitive microelectrodes and Clark-style oxygen electrodes. One pair of electrodes was moved in vertical steps through the pyramidal layer in area CA3 (moving electrode). A second stationary pair of electrodes was placed in close vicinity at ~80 µm below the slice surface (fixed electrode) to control for signal stability; (**D**) Overlay of the recording signals from the two pairs of electrodes (**black**: moving electrode, grey: fixed electrode) during bicuculline-induced ILEs. F.p., [K^+^]_o_ and oxygen drops remained stable at the fixed electrode, while f.p. amplitudes and [K^+^]_o_ peaks were larger at 100 µm compared with 0 and 200 µm at the moving electrode. Oxygen dips increased from 0 to 100 µm and remained large at a depth of 200 µm in the moving electrode; (**E**) ILE-associated amplitudes, event-related peak potassium levels and pO_2_-drops at different depths along the slice. Field potential and potassium levels showed a very similar amplitude distribution with a peak around 100 µm. Oxygen drops were smaller near the surface, but remained stable after 60–80 µm (**gray**—fixed electrode, black—moving electrode); (**F**) Fitted basal pO_2_-depth profiles (**left**, solid line) and event-associated depth profiles (**left**, dashed line) for bicuculline (**black**) and bicuculline + XE-991 (**red**). Basal OCRs (solid line, right graph) and depth-dependent event-associated OCRs (EAOCR, dotted line, right graph, EAOCR = OCR/event (mmHg·s^−1^)). Bar plots show EAOCRs for ten individual events; and (**G**) EAOCR (**top**) and ratio of EAOCR to basal OCR (**bottom**) during ILEs and SLEs. During SLEs (**red**) EAOCRs were significantly higher compared with EAOCRs during ILEs (black; *p* = 0.016, *n* = 8 and 15 for ILEs and SLEs; independent *t*-test). The ratio comparing EAOCRs to the basal OCR was significantly higher under SLEs compared with ILEs (*p* = 0.035, *n* = 8 and 15 for ILEs and SLEs, respectively, independent *t*-test).

**Figure 4:**
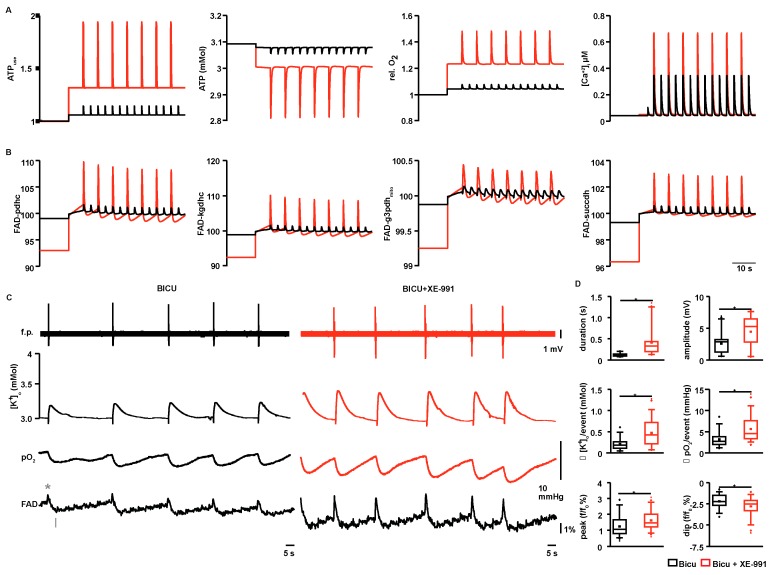
Modeling of basal and event-associated ATP consumption rates, ATP levels and FAD reduction states associated with ILEs and SLEs: (**A**) Basal ATP consumption increased by 6% and 32% compared with control during bicuculline (**black**) and bicuculline + XE-991 (**red**) application, which decreased cellular ATP levels to 3.08 mM and 3.0 mM. Event associated ATP consumption was higher under SLEs (~94% above control) compared with ILEs (~15% above control) and associated with a decrease in cellular ATP level to 3.05 mM and 2.81 mM. The third plot summarizes the relative changes in basal OCR and EAOCR (see also Figure 2B and Figure 3G). Intracellular Ca^2+^ transients are shown on the right; (**B**) FAD reduction states for FAD bound to pyruvate dehydrogenase complex (pdhc), α-ketogluterate dehydrogenase complex (kgdhc), mitochondrial glycerol-3-phosphate dehydrogenase (g3pdh_mito_), and succinate dehydrogenase (succdh). Single events elicit increased FAD oxidation in all enzymes, with a higher peak value for SLEs compared with ILEs. In order to emphasize changes in event-associated FAD redox states, ILEs and SLEs were aligned, by manually shifting the baseline; (**C**) Simultaneous f.p., [K^+^]_o_, pO_2_ and FAD-autofluorescence recording during ILEs (black) and SLEs (red). Under submerged conditions event amplitudes were smaller, yet morphologically similar to interface conditions (Figure 1 and Figure 2). Event duration, [K^+^]_o_ peaks and local pO_2_ drops increased during SLEs compared with ILEs. Simultaneously recorded FAD-transients showed increased oxidation, which was predicted by the model. Importantly, the data used for modeling was based on recordings under interface conditions; and (**D**) Quantification for C. Similar to interface conditions, SLEs showed significantly increased event durations, [K^+^]_o_-rises and local oxygen drops (**top** and **middle**). In contrast with interface conditions, f.p. event amplitudes increased from ILEs to SLEs (**top**). As predicted by the computational model, event-related FAD-transients, i.e., FAD peaks (see asterisk in **C**) and dips (see arrow in **C**), were significantly enhanced (bottom). * *p* < 0.05, *n* = 90 individual ILEs and 114 SLEs, independent *t*-test.

To calculate event-associated OCRs (EAOCRs) we quantified amplitudes of oxygen dips during individual events. Figure 3A shows exemplary field potential, [K^+^]_o_ and pO_2_ recordings for ILEs (bicuculline) and SLEs (bicuculline + XE-991). Drops in pO_2_ were significantly larger for SLEs compared with ILEs (median −31.0 mmHg vs. −5.0 mmHg, SLE: P25 −45.1, P75 −12.9, ILE: P25 −5.8, P75 −3.8, *n* = 15 (SLE) and 8 (ILE) *p* = 0.011, Figure 3B). Focal pO_2_ recordings do not reveal the location of oxygen consumption along the diffusion path between the oxygen source and the electrode location. To estimate locality of activity we measured depth-dependent field potential amplitudes and [K^+^]_o_-rises (Figure 3C–E). This revealed a bell shape of activity that was maximal at a depth of ~80–100 µm from the slice surface while pO_2_-drops per event increased successively to a depth of ~100 µm and remained elevated (no bell shape) towards the core of the slice (i.e., further away from the source of oxygen; Figure 3E). Using the spatial distribution of neuronal activity, while assuming that OCR is proportional to local neuronal activity, we calculated EAOCR for ILEs and SLEs. Figure 3F shows the mean basal OCR and EAOCR for ILEs and SLEs averaged over 8 (ILEs) and 15 (SLEs) slices and 10 events per slice. While ILEs increased local EAOCR by 3% (in absolute numbers: 0.8 mmHg·s^−1^, 0.7, 1.0) compared with basal OCR, SLEs were associated with an increased EAOCR by 18% (5.2 mmHg·s^−1^, 2.5, 10.6; Figure 3G). Compared with control condition EAOCR was elevated by 8% for ILEs and 44% for SLEs (Figure 4A, 3rd plot).

### 2.4. Modeling of ATP Consumption Rates and Prediction of FAD Transients for ILEs and SLEs

Based on OCRs and EAOCRs, we calculated ATP consumption rates and FAD transients using a model of neuronal energy metabolism [19]. Those simulations revealed a 6% and 32% increase in basal ATP demand during bicuculline and bicuculline + XE-991 compared with control conditions. Thereby, our calculations revealed a decline in cellular ATP from 3.1 to 3.08 and 3 mM (under bicuculline and bicuculline + XE-991 respectively) and FAD shifting towards a more oxidized state (Figure 4A,B). Single ILEs and SLEs increased ATP demand by 15% and 94% compared with control condition (spontaneous activity before bicuculline and/or XE-991 perfusion). Event-associated increase in ATP consumption resulted in a further drop in intracellular ATP content to 3.05 and 2.81 mM for ILEs and SLEs, respectively (Figure 4A). For four FAD containing enzymes (pyruvate dehydrogenase complex, α-ketoglutarate dehydrogenase complex, mitochondrial glycerol-3-phosphate dehydrogenase and succinate dehydrogenase), simulations predicted higher oxidation peaks and reduction shifts for SLEs than ILEs (Figure 4B). This prediction was verified experimentally by recording FAD autofluorescence of ILEs and SLEs under submerged conditions (Figure 4C). Oxidation peaks increased from median f/f_0_ of 1.1% (P25 0.8, P75 1.7, *n* = 90 events; f—event-associated fluorescence intensity, f—baseline fluorescence intensity) during ILEs to 1.5% (1.2, 2.0, *n* = 114 events, *p* < 0.05, independent *t*-test; Figure 4D bottom) for SLEs. Similarly, reduction shifts increased from −2.2% (−2.7, −1.5) to −2.5% (−3.3, −2.1, *p* < 0.05, paired *t*-test). Under submerged conditions (i.e., in the FAD imaging setup) group differences (SLEs vs. ILEs) for event duration, event-associated [K^+^]_o_ rises and oxygen drops were similar to interface conditions, but ILEs/SLEs were generally shorter (especially SLEs) and smaller in field potential, [K^+^]_o_ and pO_2_ amplitudes (Figure 4D). In contrast to interface conditions when field potential amplitudes were similar for ILEs and SLEs, under submerged conditions, amplitudes increased from 2.8 mV (1.3, 3.2) for ILEs (~half amplitude compared with interface conditions) to 5.3 mV (2.8, 6.5) for SLEs (Figure 4D).

## 3. Discussion

We studied metabolic changes and energy demand during pharmacologically induced ILEs and SLEs. We combined multiparametric recordings (field potential, [K^+^]_o_, pO_2_) in the acute hippocampal slice preparation from adult rats. By implementing pO_2_ depth profiles in theoretical models, we quantify for the first time basal OCR, EAOCR, ATP consumption, cellular ATP levels and FAD transients for ILEs and SLEs. While bicuculline did not significantly increase basal OCR, adding XE-991 mildly, but significantly, increased basal OCR. The transition from ILEs to SLEs roughly doubled the associated potassium transients, and increased EAOCR and ATP consumption by a factor of ~5.

It is well known that GABAergic disinhibition induces hypersynchronous activity in hippocampal area CA3 pyramidal neurons (e.g., [20,21]). Blockage of M-currents transforms this ILEs into SLEs [22], an observation first made in the magnesium-free seizure model [23] and confirmed by our recordings. We show that ILEs/SLEs and associated [K^+^]_o_ changes were maximal in amplitudes at a depth of ~80–100 µm (bell-shape) below the slice surface, suggesting a larger number of viable active neurons at this depth under interface conditions. In contrast, oxygen dips grew in amplitude with depth and remained elevated in the slice core despite reduced field potential amplitudes (Figure 3E). Injury during tissue preparation may explain reduced activity at the slice surface. However, in the slice core (200 µm from the surface), where oxygen levels were well above the threshold for failure in ATP production (40 mmHg) [1], a clear explanation for the reduced activity is lacking.

In the in vitro slice preparation, oxygen solely distributes by diffusion because it lacks the circulation of blood. Hence, pO_2_ cannot increase beyond a point of oxygen consumption [24]. Thus, focal oxygen recordings only reveal that oxygen was consumed between its source and the location of the electrode, but not exactly where. This may be of clinical relevance in neurointensive care patients, where cerebral blood flow (i.e., oxygen supply) is often disturbed [25], subsequently increasing the distance from the recording site to the source of oxygen. Oxygen depth-profiles recorded under interface conditions resembled those previously reported for hippocampal slices [26]. Importantly, in contrast with the whole hippocampus preparation [6], we did not observe hypoxic levels in the slice core under interface conditions. Nevertheless, reduced oxygen levels under submerged conditions may explain why ILEs/SLEs were generally shorter and smaller in field potential and [K^+^]_o_ amplitude compared with interface conditions. Oxygen-dependency (even above the hypoxia threshold) of various activity patterns, including highly energy-demanding gamma oscillation, were previously described [27]. Similarly, redox-states of NAD^+^/NAD(P)H and FAD/FADH_2_ are affected by oxygen availability. Following electrical stimulation in acute brain slices, the NADH signal is biphasic and shows prominent oxidation peaks (first phase) under 95% oxygen supply, whereas reduction overshoots (second phase) are larger when oxygen supply is reduced [26,28]. Importantly, in a recent study, FAD autofluorescence mirrored NADH signals (reduced NAD, i.e., NADH is fluorescent) indicating that either FAD or NADH signals can be used likewise [6]. In line with their findings for bicuculline-induced ictal events, we recorded prominent phases of enhanced FAD reduction following brief oxidative peaks for ILEs and SLEs in the submerged imaging setup at pO_2_ levels of ~100 mmHg [29]. Computed FAD transients based on pO_2_ recordings under interface conditions at a depth of 100 μm (i.e., with greater tissue oxygen levels of ~400 mmHg; see Figure 3F), qualitatively predicted recorded FAD signals, thereby validating the model.

Our modelling approach allowed the calculation of OCRs, ATP levels, ATP consumption rates, tricarboxylic acid cycle enzyme activities, and regulatory interdependencies that cannot be obtained directly. The extent of OCR increases during SLEs has never been determined. Kann and colleagues showed in the CA3 region in rat slice cultures a similar decrease in interstitial pO_2_ during SLEs and gamma oscillations [30]. Here we report that the transformation from interictal to seizure-like events is associated with a ca. five-fold increase in oxygen consumption. Together with the finding of increased [K^+^]_o_, greater neuronal depolarization and increased AP firing during SLEs, we propose that a major fraction of the additionally consumed oxygen during SLEs goes to Na^+^–K^+^-ATPase-mediated restoration of ion gradients and neurotransmitter reuptake and recycling [31]. Compared with control conditions (before bicuculline and/or XE-991 wash-in) the relative increase in OCR during SLEs is ~44% compared to an increase of ~8% during ILEs. This increase of OCR corresponds to an increase in ATP demand by ~94% and ~15%, respectively. This means that oxygen is more efficiently used for ATP production during SLEs than under control conditions. Oxygen is the final electron acceptor in the respiratory chain and the OCR is equivalent to respiratory chain activity. During control conditions the protons pumped by the respiratory chain are used to fuel various mitochondrial processes, e.g., ATP production, ion homeostasis, and heat production. While ATP production accounts for about 60% of the proton usage under normal conditions [32], the additional protons pumped by the respiratory chain during SLE are used almost exclusively for ATP production thereby increasing the mitochondrial energy production efficiency (ATP/O_2_). Our simulations further predicted only a moderate drop of cellular ATP levels by 9% during SLE. This is in agreement with work by Chapman et al. who measured a decrease in cellular ATP levels by 7% during short-term seizure activity [33]. This should be kept in mind when considering the role of metabolism in regulation of neuronal excitability by ATP-dependent ion channels, e.g., via ATP-sensitive potassium channels. The moderate drop in ATP levels together with the significant increase in OCR reflects the metabolic capacity of the brain to adequately increase its energy production rate during SLEs. Increased [K^+^]_o_ during ILEs/SLEs despite sufficient ATP supply thus implies that the Na^+^–K^+^-ATPase is operating at maximal velocity, yet is not able to revert massive potassium accumulation in the extracellular space during on-going epileptiform activity. The increase in ATP demand is matched by an increase in tricarboxylic acid cycle activity to produce the NADH used by the increased activity of the respiratory chain. This leads to a shift in mitochondrial redox state and an increase in the oxidized fraction of FAD in all FAD containing dehydrogenases. The return of the FAD reduction state and [K^+^]_o_ to baseline levels between successive SLEs is an additional indicator that the metabolic capacity is sufficient to meet the immediate demand as no prolonged activity of the dehydrogenases is expected. This, however, does not rule out that these types of epileptiform activity cause cellular injury in vivo. In contrast to our experimental setting where glucose and oxygen were constantly supplied, in patients following stroke or traumatic brain injury, seizures are common and may develop under conditions of impaired neurovascular coupling. Thus, shortages in energy substrates may occur due to reduced cerebral perfusion [25,34,35,36,37]. Understanding metabolic demand underlying different brain activity patterns including epileptiform activity may guide therapy, i.e., adjustment of cerebral perfusion pressure which is influenced by arterial blood pressure and the opposing intracranial pressure. In addition, knowledge about metabolic changes induced by epileptiform activity may help to detect seizure onset zones non-invasively with advanced imaging tools, such as magnetic resonance spectroscopy [38,39].

## 4. Materials and Methods

### 4.1. Slice Preparation and Maintenance

For this study, 25 male Wistar rats (weight: 200–250 g, age: 6–8 weeks) were sacrificed in accordance with the Helsinki declaration and institutional guidelines (LAGeSo Berlin, T0096/02, 2002). Animals were decapitated under anesthesia with isoflurane (2%) and laughing gas (N_2_O, 70%). Brains were rapidly removed and transferred to cold and gassed (carbogen, 95% O_2_ and 5% CO_2_) artificial cerebrospinal fluid (aCSF) containing (in mM): 129 NaCl, 21 NaHCO_3_, 10 glucose, 3 KCl, 1.25 NaH_2_PO_4_, 1.6 CaCl_2_, and 1.2 MgCl_2_. Osmolarity and pH were 295–305 mOsm/L and 7.35–7.45, respectively. Horizontal hippocampal slices (400 μm thick) were prepared with a Leica VT 1200 S vibratome (Leica, Wetzlar, Germany) and stored in an interface chamber with continuous aCSF perfusion (flow rate of 2 mL/min, temperature 34–35 °C, gassed with carbogen). Experiments started after two hours of recovery following the slicing procedure. For FAD-fluorescence imaging the slices were transferred to a submerged chamber (flow rate 10 mL/min, temperature ca. 34–35 °C) following the recovery period.

### 4.2. Electrophysiology, Oxygen Recordings and Fluorescence Recordings

All experiments were performed in area CA3 of the hippocampal formation. Simultaneous field potential and extracellular potassium ([K^+^]_o_) measurements were performed using double-barrelled ion-sensitive microelectrodes built according to the protocol of Heinemann and Arens (1992) [40]. The reference side of the electrode was filled with 154 mM NaCl while the ion sensitive side was filled with 100 mM KCl and its tip contained potassium ionophore I 60031 (Fluka, Sigma, Buchs, Switzerland). Recorded electrical potentials were converted to potassium concentrations using Nernst’s equation as previously described.

Intracellular recordings of CA3 pyramidal cells were performed simultaneously to field potential and [K^+^]_o_ recordings. We used sharp microelectrodes (resistance 60–90 MΩ) filled with 2.5 mM K^+^-acetate. Event-associated membrane depolarisations were quantified by measuring the maximal membrane depolarisation in between APs.

Basal and event-associated pO_2_ were measured using Clark-style oxygen sensors (tip: 10 μm; Unisense, Aarhus, Denmark) placed near the ion-sensitive microelectrode. Oxygen electrodes were polarized for >12 h and two point calibrated in aCSF gassed with 50% and 95% O_2_ before each recording session. For depth profiles, the pO_2_-electrode was fixed to a mechanical manipulator and moved vertically through the slice in steps of 20 μm until additional steps no longer reduced pO_2_ (usually ~200 μm below surface). To investigate the locality of neuronal activity along the slices we also performed stepwise measurements of the field potential, [K^+^]_o_ during ILEs. To exclude general signal changes, we performed this simultaneously with a second pair of electrodes placed at a depth of 80 μm (see Figure 3C). FAD^+^ (oxidized form) autofluorescence was recorded under submerged conditions using a custom-built imaging setup equipped with a light emitting diode (LED, 460 nm wavelength), a photomultiplier tube and a X20 submerged objective that was focussed on stratum pyramidale of area CA3 [41]. The LED (Lumen, Prior scientific, Seefelder, Germany) was set at 18% intensity (Power in focus plane with objective: 2.390 mW) and was triggered externally with a Master 8 (A.M.P.I., Jerusalem, Israel). To reduce bleaching and phototoxicity we performed excitation with pulsed light (5 ms, 5 Hz) [29].

### 4.3. Drugs

Epileptiform activity was induced by application of 10 μM bicuculline methiodide and subsequent co-application of 10 μM XE-991 dihydrochloride (both from Tocris, Bristol, UK).

### 4.4. Data Acquisition and Data Analysis

Analog signals were digitalized with Power1401 and recorded with Spike2 (Cambridge Electronic Design Limited, Cambridge, UK). Data analysis and statistics were performed using Spike2, Origin software (Version 6, Microcal Software, Northampton, MA, USA) and SPSS v.20.0 (IBM Corporation, Armonk, NY, USA). In most of the cases, data is shown in boxplots. Fluorescence is shown as Δf/f_0_ where f_0_ is the baseline fluorescence intensity. For ILEs and SLEs the lowest value recorded 1 second before the epileptiform discharge was set as 1. For statistical inference, we performed independent and paired Student’s *t*-tests (with Bonferroni correction when necessary) and repeated measures ANOVA. For the number of experiments, “*n*” refers to the number of slices unless stated differently. Changes were stipulated to be significant for *p* < 0.05.

### 4.5. Calculation of Basal Oxygen Consumption Rates of Control, Bicuculline and Bicuculline + XE-991 Condition

Oxygen consumption rates (OCR) were calculated from pO_2_ depth profiles measured under interface conditions as described in [18]. In short, we applied a reaction-diffusion model for oxygen consisting of diffusive oxygen transport and oxygen consumption within the slice. The slices were divided into layers with equal thickness of 1 μm. Diffusive distribution of oxygen between the layers is described by Fick’s Law with a diffusion constant of 1.6 × 10^3^ μm^2^/s [18] and oxygen consumption rate within each layer is given by Michaelis-Menten kinetics with a *K*_m_-value of 3 mmHg [42]. The OCR was assumed to be homogeneous throughout the slice (i.e., equal in every layer) and is treated as adjustable parameter to match the experimental data. Dirichlet boundary conditions were used at the slice surface and Neumann boundary conditions were used at the pO_2_ minimum. For paired measurements, pO_2_ profiles were pruned at the location of the pO_2_ minimum closest to the slice surface.

### 4.6. Calculation of Event Associated Oxygen Consumption During ILEs and SLEs

For the calculation of the OCR associated with ILEs and SLEs (event associated consumption rate, EAOCR) we took into account the locality of the events within the slice, thereby releasing the assumption of homogenous OCR. We assumed that the local OCR is proportional to the activity (magnitude of the event) in each layer of the slice. We fixed the EAOCR associated with a single event by fitting the resulting pO_2_ values at the position of the electrode to the experimental values.

### 4.7. Calculating of FAD Transients and ATP Consumption Rates

We simulated FAD transients and ATP consumption rates associated with ILEs and SLEs with a metabolic model of neuronal energy metabolism [19]. Using the OCRs obtained from pO_2_ measurements during bicuculline and bicuculline plus XE-991 under interface conditions and assuming feasible cytosolic calcium transients we determined ATP consumption rates for the different conditions by using the maximal ATP consumption rate as adjustable parameter. We validated the model prediction by comparing the predicted FAD transients with experimental data obtained during the events under submerged conditions. For all simulations we used MATLAB Release2012a (The MathWorks, Inc., Natick, MA, USA) with the optimization tool box.

## Abbreviations

aCSF	Artificial cerebrospinal fluid
ATP	Adenosine triphosphate
EAOCR	Event-associated oxygen consumption rate
FAD	Flavine adenine dinucleotide
f.p.	Local field potential
GABA	Gama-aminobutyric acid
HFO	High frequency oscillation
i.c.	Intracellular
ILE	Interictal-like event
K^+^	Potassium ion
[K^+^]_o_	Extracellular potassium concentration
LED	Light emitting diode
Na^+^	Sodium ion
NADH	Nicotinamide adenine dinucleotide
OCR	Oxygen consumption rate
pO_2_	Partial oxygen pressure
SLE	Seizure-like event
